# Successful Pregnancy in a Poor Responder: Natural In Vitro Fertilization, Menopause Treatment With Oral Contraceptives, and Anti-Müllerian Hormone as a Predictor

**DOI:** 10.7759/cureus.74650

**Published:** 2024-11-28

**Authors:** Charalampos Voros, Despoina Mavrogianni, Sofoklis Stavros, Anastasios Potiris, Dimitrios Loutradis

**Affiliations:** 1 1st Department of Obstetrics and Gynecology, National and Kapodistrian University of Athens, Medical School, Athens, GRC; 2 3rd Department of Obstetrics and Gynecology, Attikon University Hospital, National and Kapodistrian University of Athens, Medical School, Athens, GRC; 3 Department of Obstetrics and Gynecology, Fertility Institute, Assisted Reproduction Unit, Athens, GRC

**Keywords:** amh, art, infertility, ivf, premature ovarian insufficiency

## Abstract

The study focuses on spontaneous conception after menopause in a woman with primary ovarian insufficiency (POI), with an emphasis on the role of anti-Müllerian hormone (AMH) in fertility management. This case involves a 33-year-old woman with POI who has experienced both aided and spontaneous pregnancies. She had low AMH and high follicle-stimulating hormone (FSH) levels, which typically indicate a limited ovarian reserve. Despite several unsuccessful in vitro fertilization (IVF) attempts, she ultimately conceived using a modified natural cycle (MNC). Later, while on oral contraception and nearing menopause, she conceived spontaneously, resulting in a viable pregnancy. This case highlights the unpredictability of POI, the relevance of AMH as a biomarker of ovarian reserve, and the potential for spontaneous conception even after menopause, emphasizing the need for individualized reproductive treatments in managing POI patients.

## Introduction

Premature ovarian failure (POF), also known as premature menopause, is defined as the onset of amenorrhea caused by the cessation of ovarian function before the age of 40 [1]. This disorder is a significant therapeutic issue as it can lead to infertility and is associated with several long-term health problems, including osteoporosis, cardiovascular disease, and psychological effects such as anxiety and depression. Historically, high follicle-stimulating hormone (FSH) levels have been used to diagnose POF. Recently, anti-Müllerian hormone (AMH), a glycoprotein hormone secreted by the granulosa cells of ovarian follicles, has gained importance in evaluating ovarian function. AMH serves as a biomarker of ovarian reserve, reflecting the remaining number of primordial follicles [2]. It indicates ovarian age and reserve by showing how many primitive follicles are left. In clinical practice, AMH levels are now regularly evaluated to assess ovarian reserve, especially when considering infertility therapies and diagnosing POF [3].

When diagnosing POF, AMH demonstrated significantly higher sensitivity (80% vs. 28.57%) and comparable specificity (78.89% vs. 78.65%) compared to FSH. AMH is considered a more sensitive marker than FSH because it can be measured at any point during the menstrual cycle [2]. Several studies have shown that AMH levels are significantly lower in women with POF compared to healthy individuals [4]. For instance, among women with elevated FSH levels, AMH was found to be a better indicator of ovarian function than either inhibin B or antral follicle count (AFC) [5]. Due to its superior sensitivity, AMH is a valuable tool in clinical settings for monitoring ovarian reserve and guiding treatment plans for women suffering from or at risk of POF [6].

## Case presentation

In February 2018, a 33-year-old female patient presented to our reproductive facility for a comprehensive fertility assessment. The patient reported menarche at age 13 and regular menstrual cycles lasting 27 days. Her medical history included four attempts at intrauterine insemination (IUI) following a diagnostic laparoscopy conducted in February 2017. Clomiphene citrate (CC) was used in three of these attempts, while letrozole (Femara) was used in one.

A hysterosalpingogram performed in April 2015 revealed a normal uterine cavity and patent fallopian tubes. Additionally, a sperm analysis of her boyfriend showed normal parameters, ruling out male factor infertility. Early follicular phase hormone levels were measured as part of her fertility evaluation. The tested hormones included follicle-stimulating hormone (FSH), luteinizing hormone (LH), prolactin (PRL), thyroid-stimulating hormone (TSH), AMH, triiodothyronine (T3), thyroxine (T4), thyroid peroxidase antibodies (TPO), thyroglobulin antibodies (TG), and androstenedione (A). The patient’s early follicular phase FSH and AMH levels from 2015 to 2023 are presented in Table 1.

**Table 1:** Chronological Data of Reproductive Hormone Levels in the Patient From 2015 to 2023 Information on reproductive hormones arranged chronologically and with their respective units of measurement: follicle-stimulating hormone and luteinizing hormone are measured in milli-international units per milliliter (mIU/mL), E2 is measured in picograms per milliliter (pg/mL), and AMH is measured in picomoles per milliliter (pmol/mL).

Date	Follicle-stimulating hormone	Luteinizing hormone	Estradiol	Anti-Müllerian hormone
11/2015	8.8	6.6	-	-
11/2016	7.63	4.66	-	2.64
12/2017	5.6	4.4	55	1.8
05/2022	6.9	2.9	20	-
07/2023	80.2	68.32	34	-

The patient provided informed consent for her medical records to be utilized in this study, guaranteeing that her private health information would be kept private and used only for this purpose. The purpose of this extensive assessment was to determine whether there were any underlying causes of her infertility and to provide direction for creating a customized treatment program. We recommended in vitro fertilization (IVF) treatment due to her extremely low AMH levels for her age.

IVF attempts

First Attempt (May 2018)

The patient received recombinant FSH (recFSH) and a gonadotropin-releasing hormone (GnRH) analog during a brief stimulation cycle. However, this cycle was canceled on day nine since there was no follicular growth, and the rise in estradiol (E2) levels was not strong enough.

Second Attempt (June 2018)

In the second cycle, ovulation was induced using a mixture of CC, human menopausal gonadotropin (HMG), and human chorionic gonadotropin (hCG). Two mature metaphase II (MII) oocytes were collected as a consequence of this effort, and they were transferred on day three. Regretfully, the pregnancy test came back negative.

Third Attempt (September 2018)

A brief regimen including recFSH, HMG, and GnRH analogues was used in the third cycle. On day three, two oocytes were extracted and placed. The pregnancy test came out negative, just like the last time.

From March to June 2019, the patient underwent four unsuccessful modified natural cycle (MNC) IVF attempts. In March, April, and May, oocyte retrieval was successful, but no fertilization happened. A biochemical pregnancy was achieved in June but did not develop due to lowering beta hCG levels. The sixth try in September 2019 resulted in a successful pregnancy, as evidenced by growing beta hCG levels and a fetal heartbeat on ultrasound. During the patient's MNC IVF efforts in 2019, her AMH was 0.56 ng/mL (4 pmol/L), FSH ranged from 15 to 25 mIU/mL, and AFC was typically 1-2 follicles per cycle, showing difficulties in obtaining successful fertilization and pregnancy in the context of reduced ovarian reserve.

MNC attempts

Due to suboptimal outcomes from previous IVF attempts, the patient was recommended to undergo an MNC. Once the dominant follicle reached 14 mm in diameter, the MNC protocol included the injection of 200 mIU/mL of HMG, followed by daily GnRH antagonist administration. Recombinant hCG was administered, and blood levels of progesterone, LH, and E2 were tested once the follicle reached 18-19 mm. The hCG injection was given 36 hours before oocyte retrieval.

In March 2019, an oocyte was retrieved but failed to fertilize. Similar results were observed in April and May 2019. However, in June 2019, with MNC, a biochemical pregnancy was confirmed with an initial beta hCG level of 133.6 mIU/mL, which declined to 25 mIU/mL after three days. In September 2019, the patient’s beta hCG levels rose from 513.4 mIU/mL to 2210 mIU/mL in three days, indicating a viable pregnancy. An ultrasound on November 1, 2019, revealed the presence of a fetal heartbeat. On June 15, 2020, at 39 weeks of pregnancy, the patient underwent a cesarean section and delivered a healthy male baby weighing 3620 grams.

Post-delivery and after-care

The patient had regular menstrual periods until May 2023 following childbirth. However, she had not had a menstrual cycle since May when she visited us in July 2023. An ultrasound revealed no abnormalities in the ovaries and a normal uterus with an endometrial thickness of 7 mm. Menstruation did not resume after a three-month progesterone therapy regimen. Hormonal assessments suggested menopause, with LH and FSH levels at 68.32 mIU/mL and 80.2 mIU/mL, respectively. The patient was advised to take birth control pills under the guidance of an OB/Gyn expert for a period of three years. The patient returned on February 9, 2024, complaining of weariness, sickness, and vomiting. An ultrasound confirmed an eight-week fetus with a heartbeat. The patient was still taking contraceptive tablets at the time of conception, which was estimated to have happened around December 26, 2023. Amniocentesis and other appropriate diagnostic procedures were performed. The pregnancy concluded with a cesarean section at 38 weeks on September 12, 2024, and the baby weighed 2,980 grams.

## Discussion

Primary ovarian insufficiency (POI) affects one in every thousand women under the age of 30 [7]. Although a number of studies have suggested that 5-10% of women with POI may become pregnant and deliver birth (1-4%), no extensive population studies have supported this percentage [8]. Moreover, POI, also known as POF, refers to a range of decreasing oocyte quality, quantity, or reproductive capacity, with varying consequences. As a result, the frequency of spontaneous conception varies based on the particular medical circumstances [1]. The diagnostic process for POI typically involves testing FSH and estradiol levels, with recommendations for at least two random tests taken one month apart. A single FSH measurement is unreliable due to significant fluctuations, particularly in the absence of elevated FSH levels. Persistent FSH levels above 40 IU/L are generally indicative of POI [9]. The causes of POI include hereditary factors, autoimmune and enzymatic diseases, and iatrogenic consequences [10, 11]. 

The predictability of ovulation in women with POI is unclear, as hormone levels and disease activity fluctuate. This suggests that infertility in these women may not be permanent [12]. For instance, Nelson et al. discovered that 4 out of 26 women (17%) seemed to ovulate during a four-month period in a study evaluating the suppression of gonadotropins using gonadotropin-releasing hormone agonist (GnRHa), and this intervention had no effect on the result. Similarly, a study by Taylor et al. reported that 46% of women experienced at least one ovulation during a 12-week period [13]. However, in many cases, the precise cause of POI remains unknown. POI is a spectrum of decreased fertility and diminishing ovarian function that can result from an early decrease in the number of initial follicles, an increase in follicular destruction, or an insufficient response on the part of follicles to gonadotropins [14].

AMH is a crucial biomarker for predicting menopause and assessing fertility, particularly in aging populations and those delaying parenthood. AMH levels tend to decline earlier than FSH levels in cases of ovarian failure, making it a more sensitive early indicator of diminishing ovarian function. It has been demonstrated that AMH levels can predict menopause approximately five years in advance, underscoring its importance in clinical practice [15]. AMH is a more sensitive early marker for diminishing ovarian function since it declines sooner than FSH levels in cases of ovarian failure [12]. Several studies have linked AMH levels and AMH receptor polymorphism with the ovarian response of women in IVF protocols, the outcome of IVF attempts, and other conditions related to a woman’s physiology and pathology [16]. AMH studies that cover a broad age range (20-49 years) improve the model's suitability for younger women and increase the menopause onset prediction by multiple decades. Over the course of a six-year research period, Sowers et al. saw a steady drop in AMH levels across all age groups [14]. Studies have explored the impact of genetic polymorphisms, such as those in the AMH receptor (AMHRII) and FSH receptor (FSHR), on IVF outcomes. For instance, carriers of certain polymorphisms may have higher basal FSH levels but also greater AMH levels, suggesting a potential role in tailoring IVF treatments [17]. 

Research examined the distribution of AMHRII -482A>G and FSHR Ser680Asn polymorphisms in 126 infertile women versus 100 fertile women. The results showed no significant difference in genotype frequencies between the groups, nor was there a link between polymorphism combinations and infertility. However, in a subgroup of women who had undergone more than two IVF attempts in the past, carriers of the polymorphism had significantly higher basal FSH levels compared to non-carriers [18]. In a different investigation, serum and follicular fluid AMH levels, as well as the FSHR Ser680Asn polymorphism, were examined in 32 IVF/ICSI-undergoing women. The study observed that women carrying the Ser allele had more follicles and oocytes, but it did not find any significant changes in AMH levels across genotypes. Women with AMH levels below 2.22 ng/mL had lower serum and follicular fluid AMH ratios [3]. In another study, 151 infertile women and 100 healthy controls were examined for AMH and AMHRII SNPs (Ile49Ser and -482A>G). Women with one polymorphism have, on average, 5.5 units higher levels of AMH compared to women carrying no polymorphism, while for women with no polymorphisms, each unit of FSH increase is associated with a 72% lower average concentration of blood AMH. All of these findings suggest the possibility that AMH levels and genetic polymorphisms, like FSHR and AMHRII, might direct customized therapy during IVF/ICSI procedures [19].

In our case, the patient’s history included: (1) a significantly elevated basal serum FSH concentration of 80.1 mIU/mL, based on a single test; (2) a noticeably low basal estrogen level of 34 pg/mL; (3) a low AMH level recorded at 0.56 ng/mL; and (4) ultrasonography revealing the absence of antral follicles in both ovaries. These clinical features formed the basis for diagnosing POI in this report. Taken together, these criteria provide a solid foundation for the diagnosis of POI. A prolonged absence of ovarian activity is indicated by chronic abnormal menstrual periods, and the increased FSH level suggests the pituitary gland's attempt to activate the ovaries through feedback. The diagnosis is further supported by the low levels of AMH and estrogen, indicating reduced ovarian reserve and function [17]. Visual confirmation of the absence of active ovarian follicles is provided by the lack of antral follicles on ultrasonography, consistent with POI. The patient’s low AMH levels prompted a recommendation for IVF, with expectations of menopause within six years [14]. The patient underwent successful assisted reproduction in 2020 and had reached menopause by 2024, aligning with the anticipated timeline based on her low AMH levels.

The patient had normal menstrual periods after giving birth until reaching menopause at age 38 and starting contraceptive pills. Nevertheless, ovulation and pregnancy occurred spontaneously. One possible explanation for the reported spontaneous pregnancy during the menopausal phase could be the use of contraceptive tablets. Although the exact process causing this phenomenon is unknown, two theories have been proposed. The Exogenous Estrogen Hypothesis suggests that exogenous estrogens may improve the ovarian response to endogenous gonadotropins and restore ovarian receptor sensitivity. Exogenous estrogens might enhance follicle-stimulating hormone (FSH) receptor sensitivity and promote follicle recruitment by normalizing increased gonadotropin levels [20]. The Genetic Heterogeneity Hypothesis suggests that certain women may be more susceptible to the effects of hormone therapies due to incomplete penetrance genetic differences in the FSH receptor or regulatory enzymes. These genetic variants may elevate intracellular cAMP levels and improve hormone-sensitive adenylate cyclase function.

In this case, the patient exhibited typical POI features, including significantly elevated FSH, low estrogen, low AMH, and the absence of antral follicles on ultrasonography. These findings provided a strong basis for diagnosing POI. Interestingly, the patient conceived spontaneously despite being in the menopausal phase and using contraceptives. This phenomenon could be explained by two hypotheses: the Exogenous Estrogen Hypothesis, suggesting enhanced ovarian response to endogenous gonadotropins due to exogenous estrogens, and the Genetic Heterogeneity Hypothesis, which posits that genetic variations in hormone receptors or enzymes may increase sensitivity to hormonal therapies.

AMH serves as a key biomarker for predicting menopause, particularly in younger women. While IVF protocols may not be effective for all patients, MNCs offer a viable alternative. Additionally, the potential for spontaneous pregnancy during premature menopause, possibly influenced by contraceptive use, highlights the complexity of POI and the importance of personalized reproductive strategies.

## Conclusions

AMH is a key biomarker for predicting menopause, particularly in younger women. MNCs present a potential alternative for people who do not respond well to standard IVF methods. Furthermore, although the mechanism is not well understood, spontaneous pregnancy can occur after premature menopause while using contraceptives.
